# Genome-wide characterization of *COMT* family and regulatory role of *CsCOMT19* in melatonin synthesis in *Camellia sinensis*

**DOI:** 10.1186/s12870-023-04702-0

**Published:** 2024-01-16

**Authors:** Thanh Huyen Pham, Xingyu Tian, Huimin Zhao, Tong Li, Litang Lu

**Affiliations:** 1 College of Life Science, The Key Laboratory of Plant Resources Conservation and Germplasm Innovation in the Mountainous Region (Ministry of Education), Guizhou University, Guiyang, 550025 People’s Republic of China; 2 College of Tea Science, Guizhou University, Guiyang, 550025 People’s Republic of China

**Keywords:** COMT, *Camellia sinensis*, Gene evolution, Gene expression, Biotic and abiotic stresses, Melatonin synthesis

## Abstract

**Background:**

Caffeic acid O-methyltransferase (COMT) is a key enzyme that regulates melatonin synthesis and is involved in regulating the growth, development, and response to abiotic stress in plants. Tea plant is a popular beverage consumed worldwide, has been used for centuries for its medicinal properties, including its ability to reduce inflammation, improve digestion, and boost immune function. By analyzing genetic variation within the *COMT* family, while helping tea plants resist adversity, it is also possible to gain a deeper understanding of how different tea varieties produce and metabolize catechins, then be used to develop new tea cultivars with desired flavor profiles and health benefits.

**Results:**

In this study, a total of 25 *CsCOMT* genes were identified based on the high-quality tea (*Camellia sinensis*) plant genome database. Phylogenetic tree analysis of CsCOMTs with COMTs from other species showed that COMTs divided into four subfamilies (Class I, II, III, IV), and CsCOMTs was distributed in Class I, Class II, Class III. *CsCOMTs* not only undergoes large-scale gene recombination in pairs internally in tea plant, but also shares 2 and 7 collinear genes with *Arabidopsis thaliana* and poplar (*Populus trichocarpa*), respectively. The promoter region of *CsCOMTs* was found to be rich in *cis*-acting elements associated with plant growth and stress response. By analyzing the previously transcriptome data, it was found that some members of *CsCOMT* family exhibited significant tissue-specific expression and differential expression under different stress treatments. Subsequently, we selected six *CsCOMTs* to further validated their expression levels in different tissues organ using qRT-PCR. In addition, we silenced the *CsCOMT19* through virus-induced gene silencing (VIGS) method and found that *CsCOMT19* positively regulates the synthesis of melatonin in tea plant.

**Conclusion:**

These results will contribute to the understanding the functions of *CsCOMT* gene family and provide valuable information for further research on the role of *CsCOMT* genes in regulating tea plant growth, development, and response to abiotic stress.

**Supplementary Information:**

The online version contains supplementary material available at 10.1186/s12870-023-04702-0.

## Background

Melatonin, chemically named N-acetyl-5-methoxytryptamine, is a highly conserved small-molecule tryptophan indole derivative. Melatonin and its metabolites have attracted attention due to their ability to continuously scavenge reactive oxygen species (ROS) or reactive nitrogen species (RNS) [1]. Study showed that one molecule of melatonin can scavenge up to ten ROS or RNS molecules, thus even lower concentrations of melatonin can effectively protect organisms against oxidative stress [2, 3]. Exogenous application of melatonin could slow down senescence caused by various abiotic stresses and regulate physiological processes such as circadian rhythm, explant growth and flowering, and seed germination as a multifunctional signaling molecule [4–9].

In recent years, research has found that plant melatonin is mainly regulated by six different enzymes, including tryptophan decarboxylase (TDC), tryptophan hydroxylase (TPH), tryptamine 5-hydroxylase (T5H), serotonin N-acetyltransferase (SNAT), acetylserotonin methyl transferase (ASMT), and caffeic acid O-methyltransferase (COMT) [4]. Among them, COMT and ASMT that belong to the O-methyltransferase family can methylate phenylpropane compounds, flavonoids, and alkaloids [10]. COMT has been found in various species such as *Arabidopsis thaliana* [11], rice (*Oryza sativa*) [12], longan (*Dimocarpus longan*) [13], etc. COMTs catalyze N-acetyl serotonin into melatonin [10, 14]. The overexpression of them also can help plant grow [15]. Sorghum bicolor *COMT* can be involved in tricin biosynthesis methylated the flavones luteolin and selgin [16]. The expression of *COMT4* in aspen can change the structure of lignin, which increase the crosslinking of condensed lignin subunits by G-units [17]. However, little information is available about the *COMT* genes and their functions in tea plants.

Tea plant (*Camellia sinensis*) is an important economic crop, and non-alcoholic beverages made from its buds and leaves are popular worldwide [18, 19]. Tea contains many health benefits such as aiding digestion, antibacterial effects, reducing blood pressure, sugar and cholesterol, antioxidant effects, and etc. due to its high content of bioactive catechins, vitamins, polyphenols, flavonoids, and medicinal properties [20–22]. As a plant that thrives in warm and humid environments, tea plants are often subjected to various external stresses during their growth process, such as drought and low temperature stress, pest and disease infestations, which can significantly reduce the growth, development, quality, and yield of tea leaves. Due to environmental changes, problems caused by abiotic stresses such as drought and low temperature have become increasingly prominent, leading to significant reductions in tea production and quality and causing enormous economic losses [23]. Therefore, exploration of the mechanisms underlying tea plant stress tolerance and in-depth mining of stress-related genes are of great theoretical and practical significance.

In this study, we conducted a whole-genome identification of the *CsCOMT* family in tea plant using bioinformatics methods based on the high-quality tea plant genome database. A total of 25 *CsCOMT* genes were identified, and the physicochemical properties, gene structures, and subcellular localization were predicted. The structural characteristics and evolutionary traits of the tea plant *CsCOMT* family were clarified. Simultaneously, we also analyzed the expression patterns of 25 *CsCOMT* genes in different tissues and under stress based on the transcriptome data. In addition, the virus-induced gene silencing (VIGS) approach was used to investigate the effect of the *CsCOMT19* on melatonin content. This study aims to provide reference for revealing the potential functions of *CsCOMT* family members in tea plants.

## Results

### Identification of the *CsCOMT* family genes in tea plant

Using the amino acid sequence of *Arabidopsis COMT* as a BLAST query, we identified 25 candidate tea plant *COMT* genes (*CsCOMTs*) from the tea plant genome database. Then, these candidate genes were submitted to online websites for structural validation of conserved domains through two rounds of comparison search. Finally, all 25 *CsCOMT* genes were confirmed to belong to the *COMT* family and named based on their homology with Arabidopsis *COMT* and their position on chromosomes.

Through the analysis of the physicochemical properties of members of the *CsCOMT* gene family, it was found that the CsCOMT proteins had an amino acid range of 186 (CsCOMT25) to 393 (CsCOMT13); the relative molecular weight was between 20.73 (CsCOMT25) to 43.80 kD (CsCOMT13); the theoretical isoelectric point (*pI*) ranged from 4.94 (CsCOMT1) to 6.49 (CsCOMT25). It was found that all of 25 CsCOMT proteins have a *pI* < 7, indicating that most CsCOMT proteins are rich in acidic amino acids. Most subcellular localizations of CsCOMT family members were in chloroplasts, among which CsCOMT5 was located in mitochondria, CsCOMT13 was located in both chloroplasts and mitochondria, and CsCOMT25 was located in the nucleus (Table 1).

**Table 1:** Information of *CsCOMT* genes identified in *C. sinensis*

**Gene name**	**Accession No**	**Intron**	**Chromosomal location**			**Protein Properties**			**Subcellular location**
			**Chr**	**Start**	**End**	**Length (aa)**	**MW (kDa)**	***pI***	
*CsCOMT1*	CSS0039718.1	4	3	92,432,006	92,437,542	371	40.17	4.94	Chloroplast
*CsCOMT2*	CSS0037946.1	3	3	91,956,040	91,961,165	368	39.87	5.14	Chloroplast
*CsCOMT3*	CSS0048493.1	3	1	112,085,937	112,091,308	351	38.32	5.94	Chloroplast
*CsCOMT4*	CSS0037320.1	3	3	92,351,303	92,356,400	368	39.85	5.14	Chloroplast
*CsCOMT5*	CSS0017751.1	3	8	139,036,103	139,042,539	391	42.92	5.76	Mitochondrion
*CsCOMT6*	CSS0036494.1	3	9	56,007,566	56,012,718	366	40.16	5.95	Chloroplast
*CsCOMT7*	CSS0007434.1	1	5	94,698,511	94,711,799	354	39.10	5.45	Chloroplast
*CsCOMT8*	CSS0006936.1	1	5	97,659,841	97,664,938	355	39.16	5.60	Chloroplast
*CsCOMT9*	CSS0035451.1	2	7	59,409,307	59,412,279	358	40.53	5.55	Chloroplast
*CsCOMT10*	CSS0025746.1	2	2	106,494,711	106,504,385	319	34.95	5.64	Chloroplast
*CsCOMT11*	CSS0029228.1	1	7	55,144,050	55,147,115	357	39.60	5.57	Chloroplast
*CsCOMT12*	CSS0003573.1	2	5	111,181,605	111,186,182	346	38.95	5.87	Chloroplast
*CsCOMT13*	CSS0036567.1	1	7	55,209,520	55,211,648	393	43.80	6.06	Mitochondrion
*CsCOMT14*	CSS0042551.1	1	7	54,550,266	54,552,772	357	39.62	5.57	Chloroplast
*CsCOMT15*	CSS0015347.1	1	6	171,279,383	171,282,903	347	38.85	5.47	Chloroplast
*CsCOMT16*	CSS0033599.1	1	6	171,235,170	171,238,679	347	38.85	5.47	Chloroplast
*CsCOMT17*	CSS0000080.1	1	6	171,195,877	171,200,372	347	38.88	5.47	Chloroplast
*CsCOMT18*	CSS0006416.1	1	3	1,899,577	1,901,052	350	38.66	5.49	Chloroplast
*CsCOMT19*	CSS0044619.1	1	5	111,082,799	111,087,666	340	38.50	5.88	Chloroplast
*CsCOMT20*	CSS0023656.1	3	7	54,505,207	54,507,188	324	36.28	6.00	Chloroplast
*CsCOMT21*	CSS0003281.1	2	5	110,166,060	110,167,265	295	32.28	5.24	Chloroplast
*CsCOMT22*	CSS0037428.1	1	2	107,557,035	107,557,800	244	27.02	6.19	Chloroplast
*CsCOMT23*	CSS0018617.1	1	5	97,874,374	9,787,139	244	26.98	6.40	Chloroplast
*CsCOMT24*	CSS0033028.1	1	5	95,030,304	95,031,069	244	26.88	6.19	Chloroplast
*CsCOMT25*	CSS0035899.1	0	3	46,478,866	46,479,426	186	20.73	6.49	Nucleus

### Phylogenetic analysis and conserved motifs of CsCOMTs

**Fig. 1:**
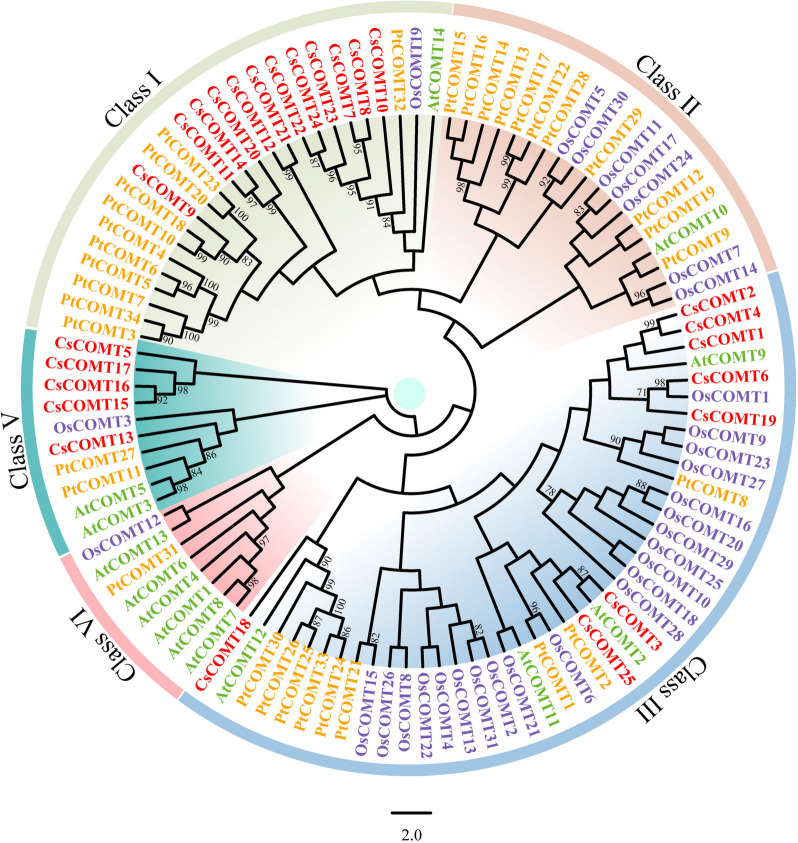
Phylogenetic analysis of COMTs identified in tea plants, *Arabidopsis*, poplar, and rice. Different colors represent different groups, and different abbreviations represent COMTs from tea plant (CsCOMT), *Arabidopsi*s (AtCOMT), poplar (PtCOMT), and rice (OsCOMT)

To clarify the classification of the CsCOMT family members, multiple sequence alignments were performed using CsCOMTs and COMTs from *Arabidopsis*, rice, and poplar. The results showed that CsCOMTs had high homology with COMTs from other species. According to the analysis of the evolutionary relationships among tea plants, *Arabidopsis*, rice, and poplar, the members of the COMT family were divided into four subfamilies (Fig. 1, Fig. S1). The members of the tea tree CsCOMT family are distributed in Class I, Class III, and Class V. Among them, CsCOMT has the highest number of members in Class I (12 members) (Fig. S2).

**Fig. 2:**
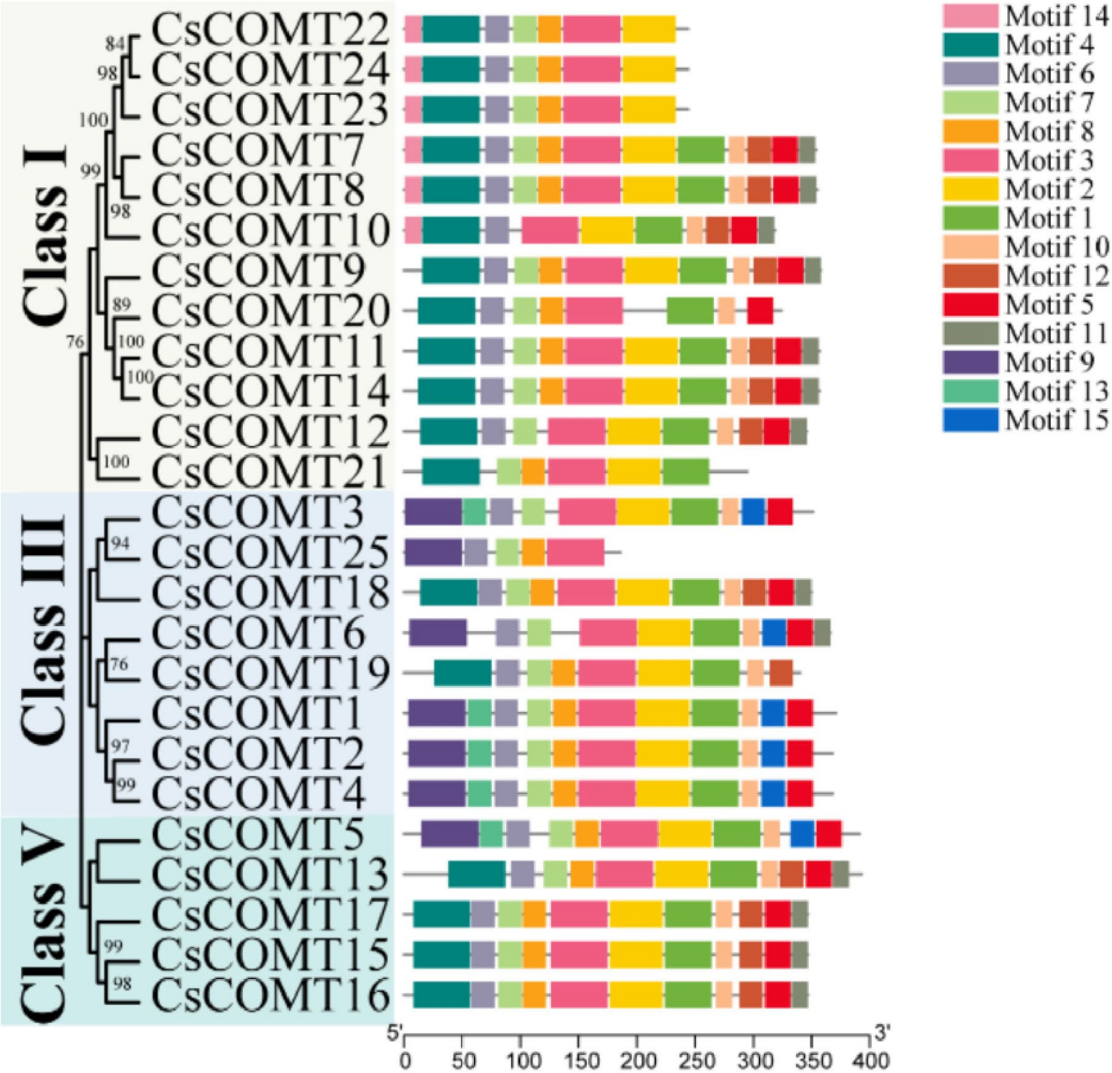
The conserved motifs of CsCOMTs

Further analyzed the conserved motifs of CsCOMT family members, and the results showed that CsCOMT family members contained a large number of co-existing motifs, including motif 3 (Fig. 2, Fig. S3). The type of conserved motifs in each subfamily were basically the same, indicating that the same subfamily was composed of similar conserved structural domains and may have similar biological functions.

### Chromosomal localization and gene structure of *CsCOMT* members

**Fig. 3:**
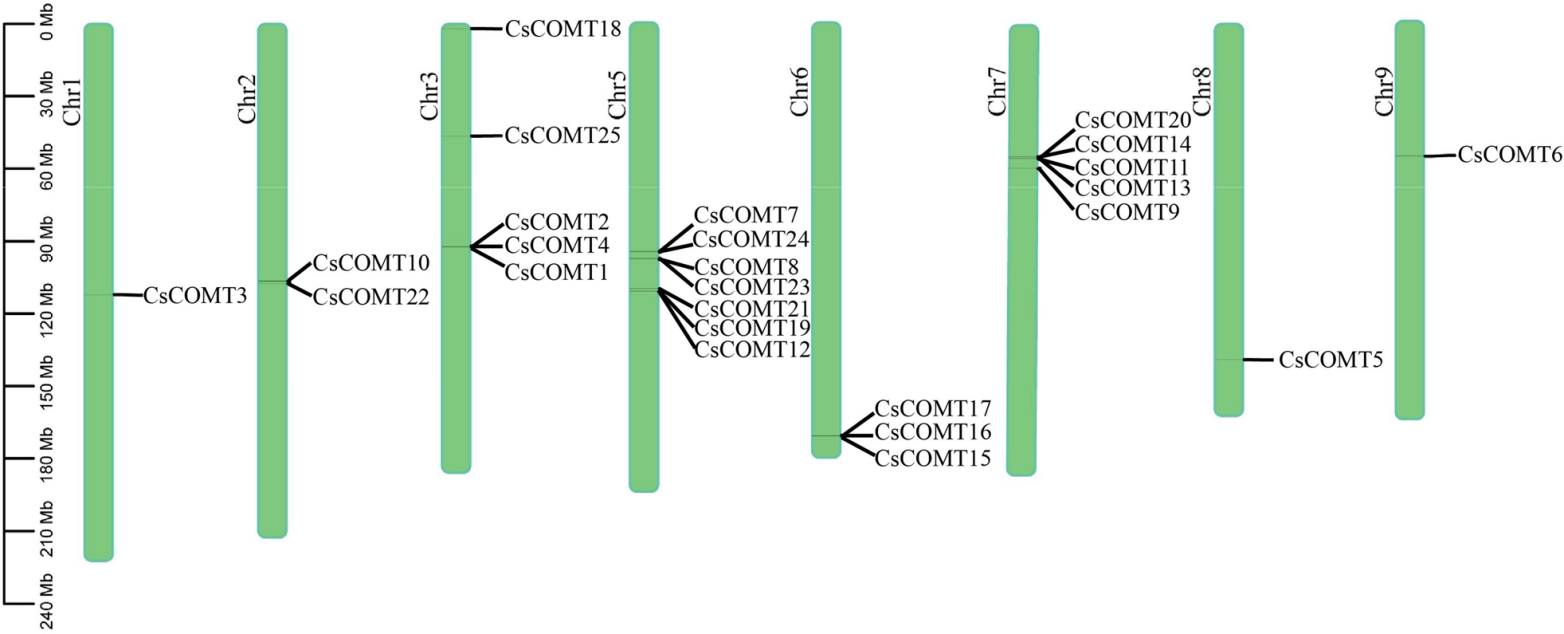
The chromosome localization of *CsCOMT* genes

**Fig. 4:**
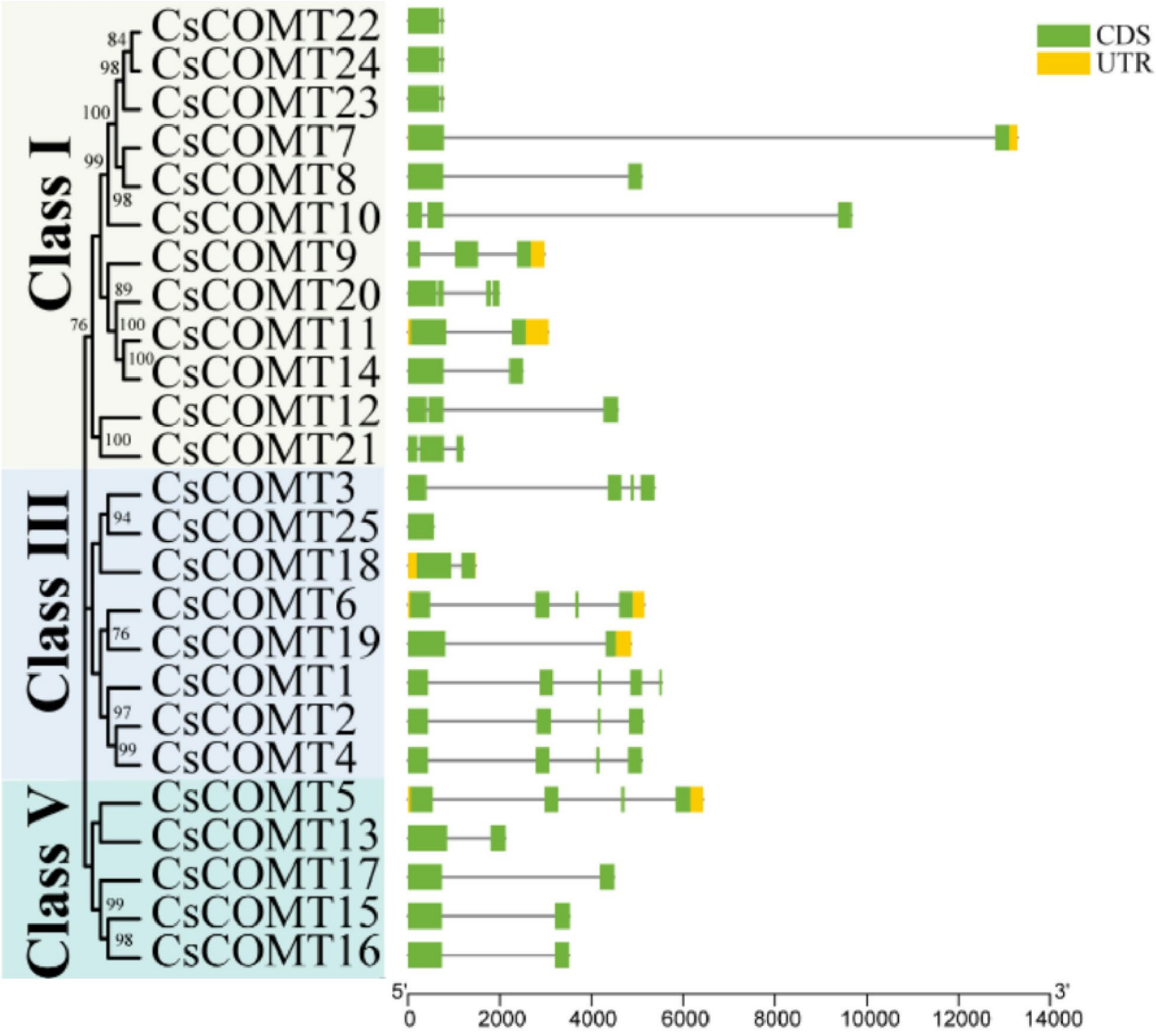
Structures of the 25 *CsCOMT* genes

The members of *CsCOMT* family were most distributed on chromosome 5, with seven members, while chromosomes 1, 8, and 9 were the least distributed, with one member on each chromosome (Fig. 3, Table S1). In order to gain a deeper understanding of the *CsCOMT* family genes function, the structure of *CsCOMTs* were analyzed. By analyzing the sequence composition of the *CsCOMT* gene, it was found that all *CsCOMTs* in tea plants contain introns. From the distribution of UTR, we can see that the UTR of *CsCOMT* gene family is relatively small (Fig. 4).

**Fig. 5:**
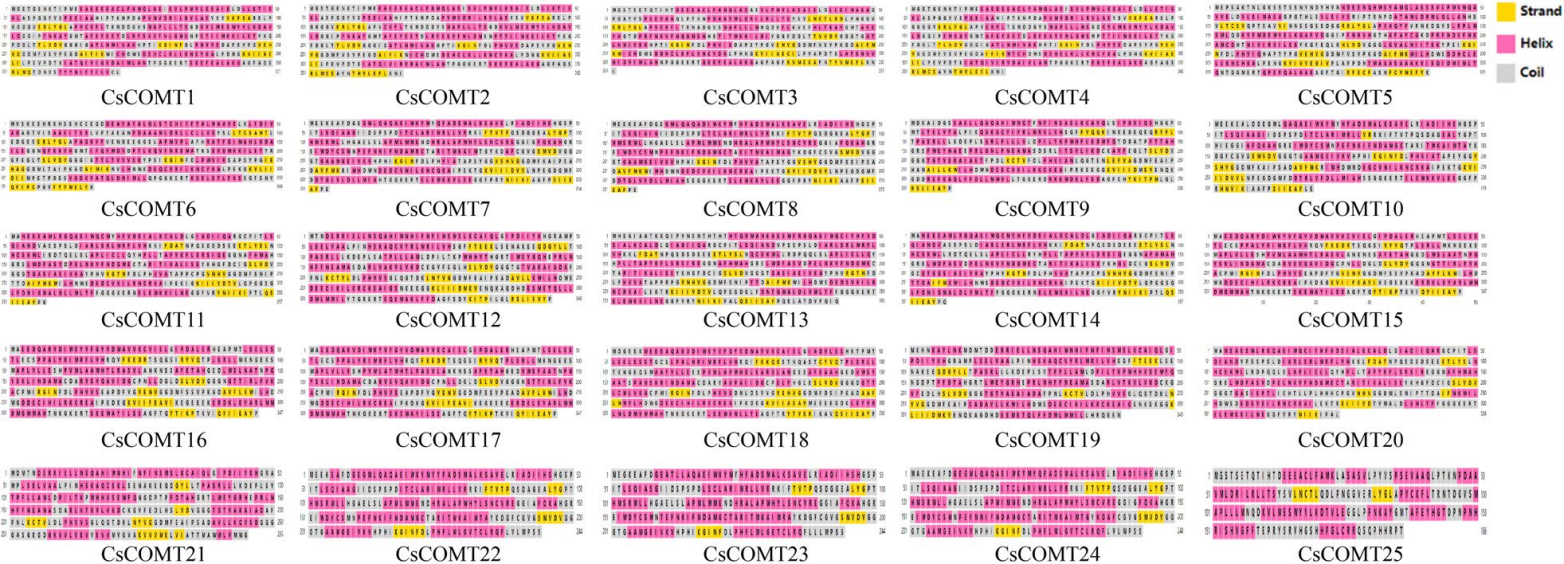
Secondary structure of CsCOMT family members

**Fig. 6:**
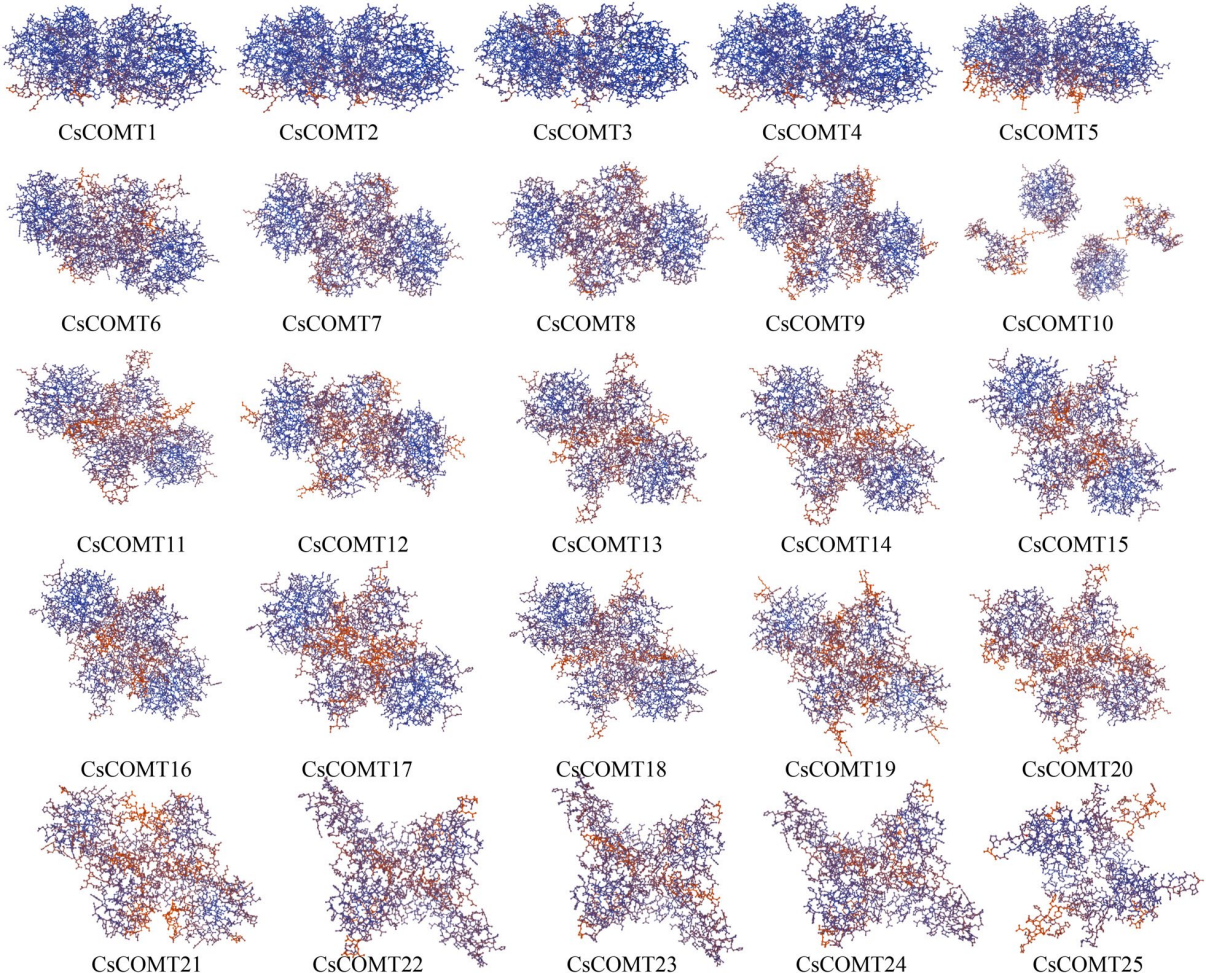
Tertiary structure of CsCOMT family members

Further analysis of the secondary structure of the *CsCOMT* family members in tea plants revealed that *CsCOMT* is mainly composed of helix, followed by coil, and finally strand (Fig. 5). Overall, these three structures are intertwined. Subsequently, protein tertiary structure prediction was performed, and it was found that the tertiary structures of CsCOMTs was mostly single-cluster shape, expect that CsCOMT10 showed a double-cluster distribution. In addition, CsCOMT1, CsCOMT2, CsCOMT3, CsCOMT4, and CsCOMT5 exhibited different shapes from other family members (Fig. 6).

### Collinearity analysis

**Fig. 7:**
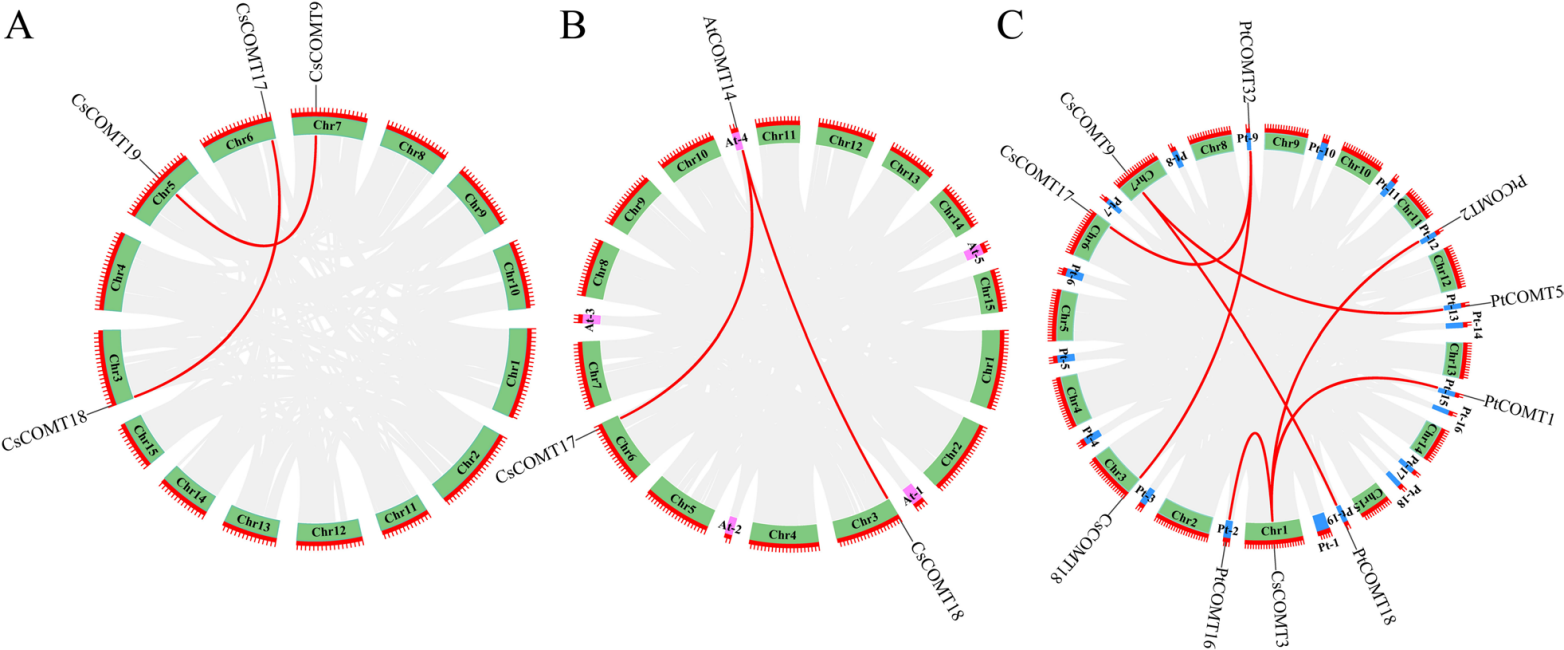
The collinearity analysis of *CsCOMT*s. **A** Large-scale duplication analysis of *CsCOMT*s; **B** Large-scale duplication analysis of *COMT*s between tea plants and *Arabidopsis*; **C** Large-scale duplication analysis of *COMT*s between tea plants and poplar

Through collinearity analysis, it was found that there was a large segment duplication among members of the *CsCOMT* family, including *CsCOMT17* and *CsCOMT18*, *CsCOMT19* and *CsCOMT9*. Subsequently, we conducted a large segment recombination analysis between tea plant and *Arabidopsis* and found that *CsCOMT17*, *CsCOMT18*, and *AtCOMT14* underwent large segment recombination. In addition, many large segment recombination events were also observed between tea plant and poplar, including *CsCOMT3*, *CsCOMT18*, *CsCOMT17*, and *CsCOMT9*, and *PtCOMT32*, *PtCOMT2*, *PtCOMT5*, *PtCOMT1, PtCOMT18*, and *PtCOMT16* (Fig. 7). In summary, the *CsCOMT* family members undergo large segment duplications within themselves, as well as with other species, meaning that some family members share homologous segments with those from other species.

### The *cis*-acting elements predicted to be present in *CsCOMTs* promoters

**Fig. 8:**
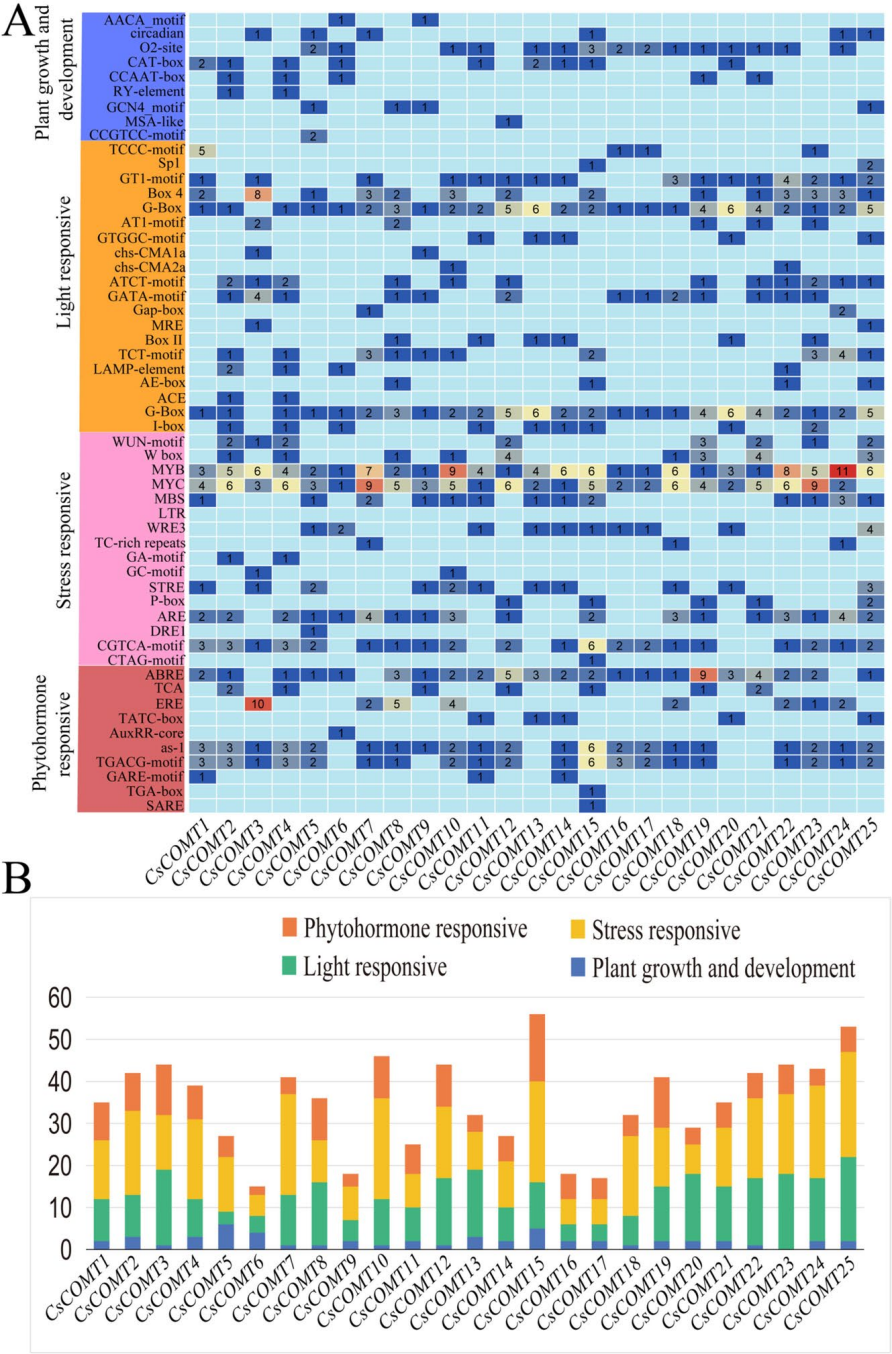
Analysis of *cis*-acting elements in the promoter region of *CsCOMTs.* (**A**) The left figure represents the evolutionary lineage of members of the tea plant *COMT* family, while the right figure represents the composition of homeostatic elements in the tea plant *COMT* family. (**B**) Distribution of cis-acting elements with related functions in *CsCOMTs* family

To explore the putative biological functions of *CsCOMT* members**,** the *cis*-acting elements in the promoter regions of *CsCOMTs* were analyzed. As shown in Fig. 8A and B, the *CsCOMTs* promoters contained various enriched elements, which mainly include four aspects: plant growth and development, light response, stress response, and hormone response. Many *CsCOMT* genes contain many types of homeopathic elements, such as *CsCOMT12*, *CsCOMT21*, *CsCOMT7*, *CsCOMT24*, *CsCOMT19*, etc. Among them, *CsCOMT19* contains 5 types related to growth and development (CAAT-box, CAT-box, CCAAT-box, O2-site, and motif I); There are 6 categories related to photoreaction (chs-CMA1a, chs-CMA2a, G-Box, ATCT-motif, AT1-motif, and Box4); There are 6 types related to stress (MYC, W-box, MYB, P-Box, LTR, and WRE3); There is one type of hormone related (TGACG-motif).

### GO functional enrichment analysis

**Fig. 9:**
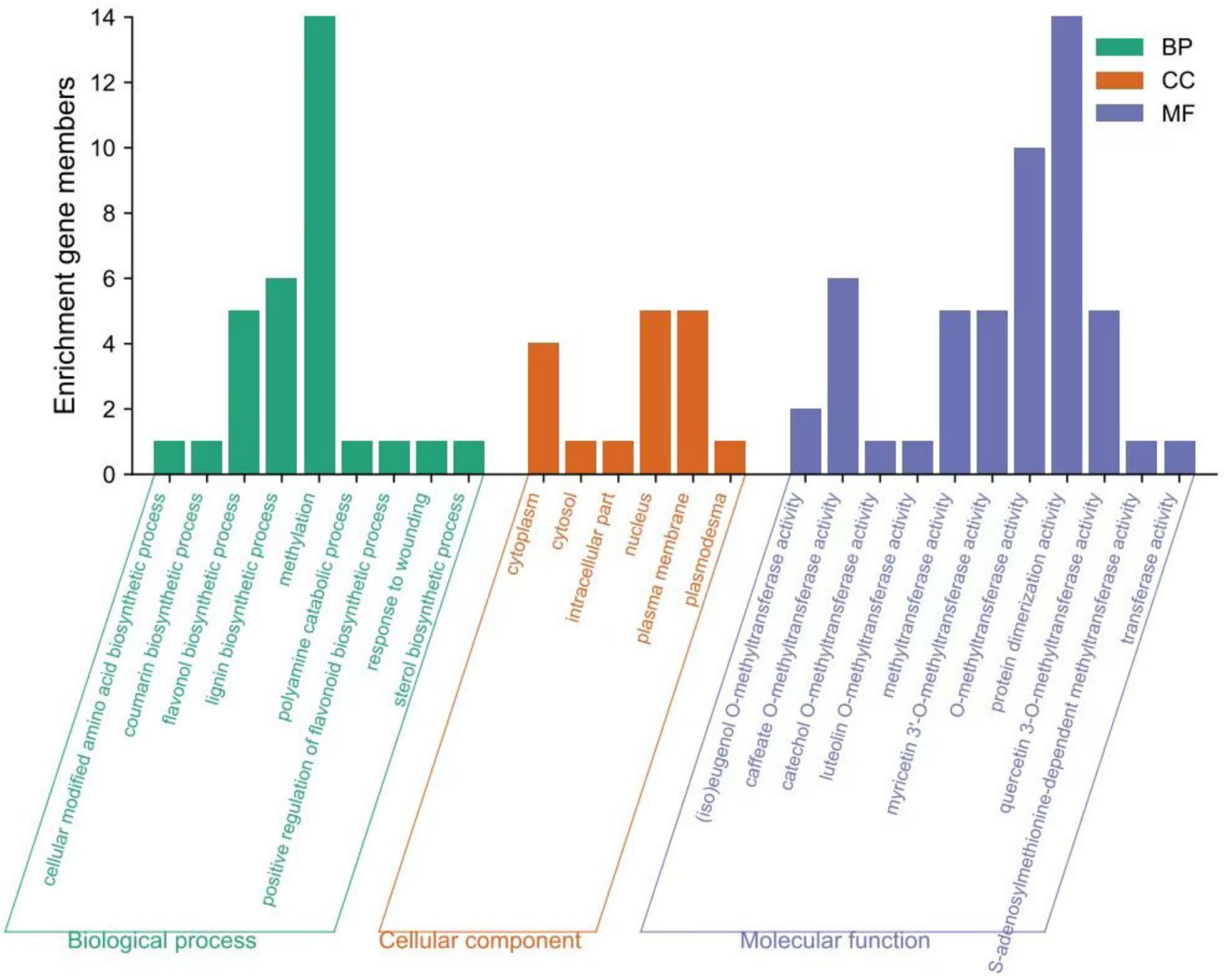
CsCOMT functional enrichment analysis

In order to further elucidate the biological functions of the CsCOMT gene family, we conducted GO enrichment analysis on its members (Fig. 9). The members of the COMT family of tea plants are mainly enriched in the flavonol biosynthetic process, lignin biosynthentic process, and metabolism in biological processes; The main enriched cellular components are cycloplasm, nucleus, and plasma membrane; Molecular functions are mainly enriched in buffered O-methyltransferase activity, methyltransferase activity, myricetin 3 '- O-methyltransferase activity, O-methyltransferase activity, protein dimerization activity, and quercetin 3-O-methyltransferase activity.

### Transcriptome data analysis

**Fig. 10:**
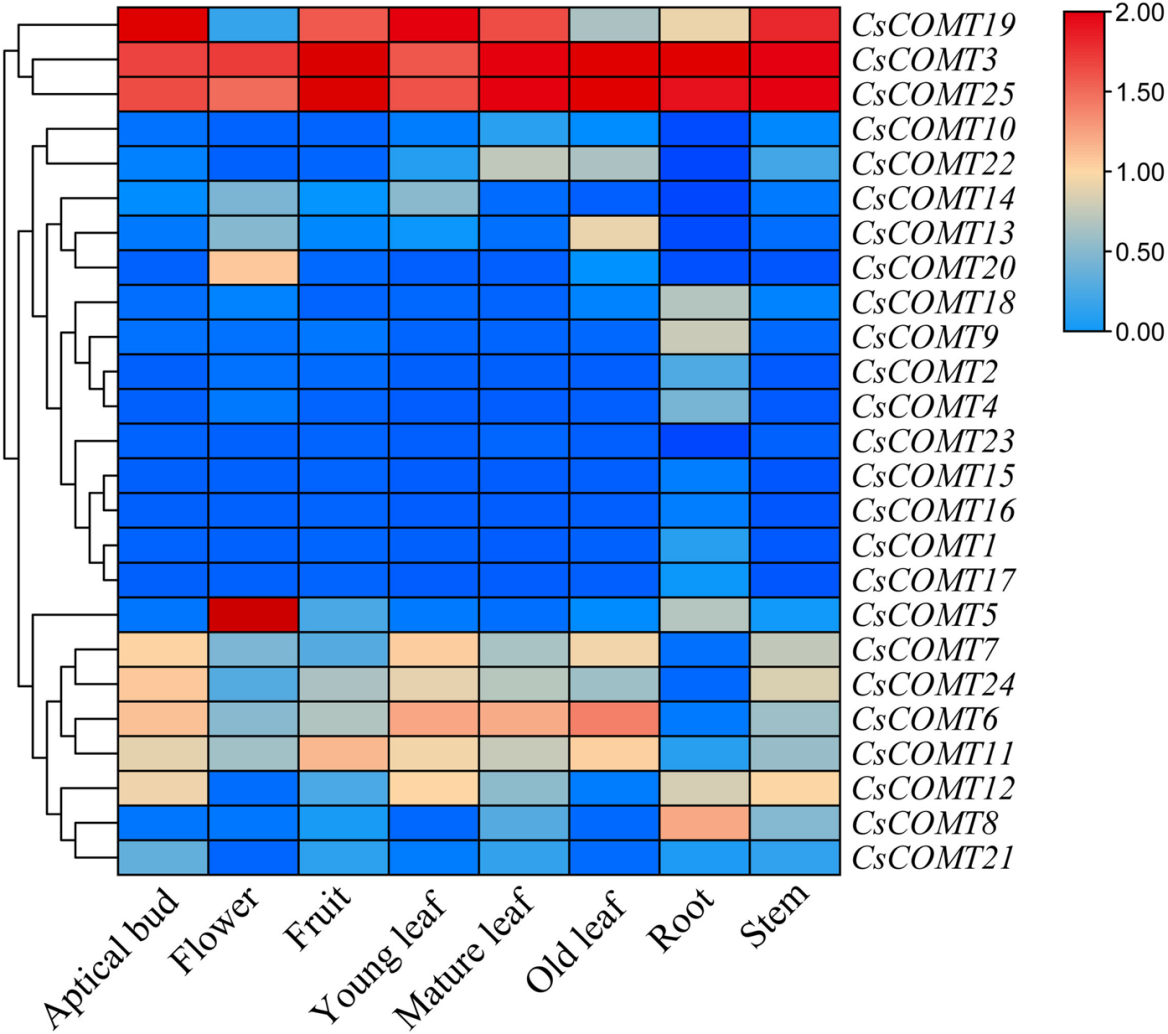
Expression of *CsCOMTs* in different tissues of tea plants

We used previously published transcriptome dataset to analyze the expression patterns of 25 *CsCOMTs* in different tissues and under different stress conditions. As shown in Fig. 10, some *CsCOMTs* were not expressed in multiple tissues, such as *CsCOMT20* only expressed in flowers, *CsCOMT18*, *CsCOMT9*, *CsCOMT2*, *CsCOMT4*, *CsCOMT1*, and *CsCOMT17* only expressed in roots. While some *CsCOMTs* had high expressions in various tissues (stems, young leaves, mature leaves, old leaves, flowers, fruits, and young buds), such as *CsCOMT19*, *CsCOMT3*, *CsCOMT25*, *CsCOMT11*, *CsCOMT6*, *CsCOMT24*, *CsCOMT12*, *CsCOMT22* and *CsCOMT18*. In addition, some *CsCOMTs* were also expressed in a few tissues, such as *CsCOMT5* in stems, roots, and flowers; *CsCOMT14* in young leaves and flowers; *CsCOMT13* in old leaves and flowers. These results indicate that different *CsCOMT* genes may be involved in different growth and developmental processes of tea plants.

**Fig. 11:**
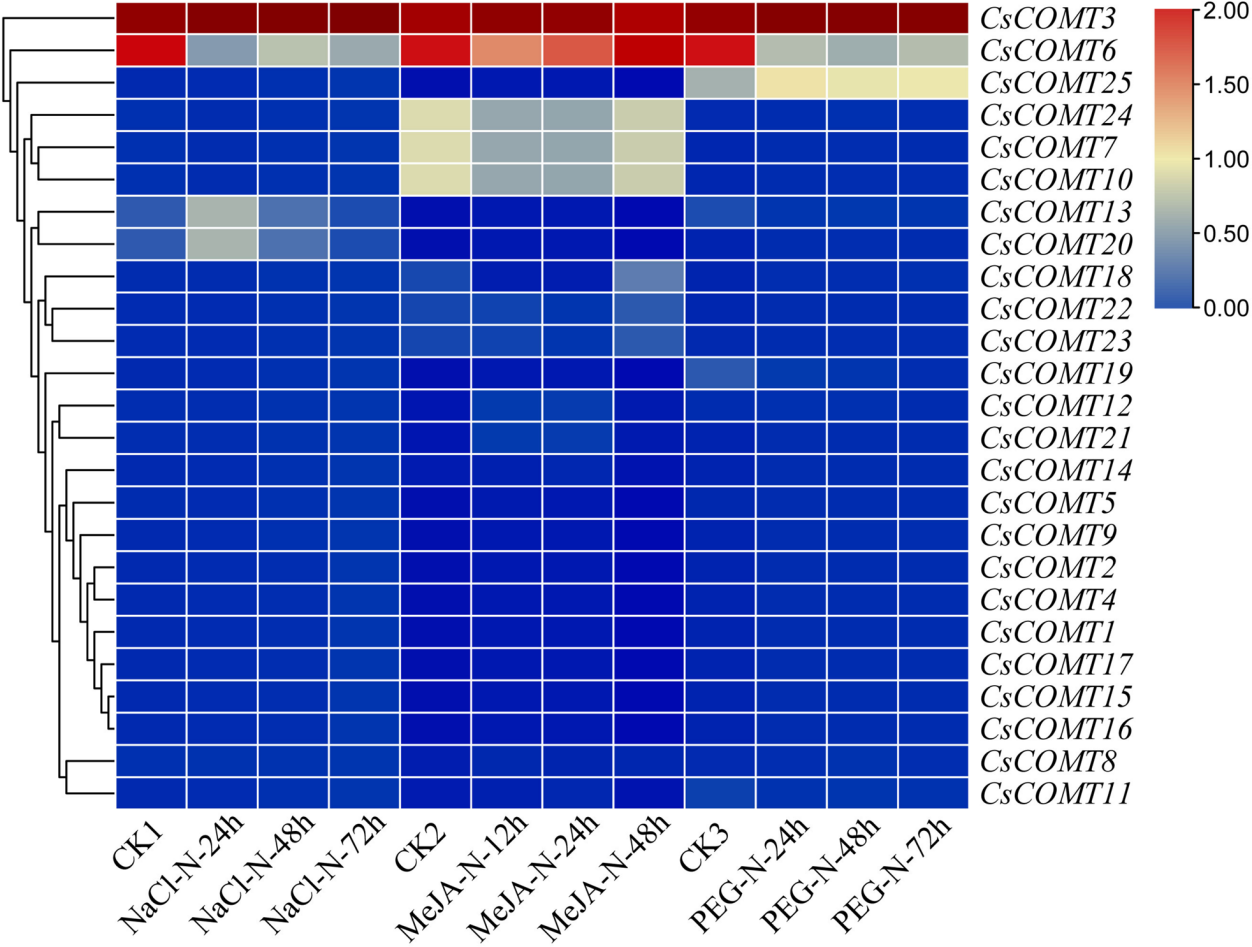
Expression of *CsCOMTs* under different stress conditions

Under different stress conditions (salt stress, drought stress), the expression of *CsCOMTs* were various (Fig. 11). Among them, the expression level of *CsCOMT6* gradually decreased with the prolongation of salt stress time, while the expression levels of *CsCOMT13* and *CsCOMT20* first increased and then decreased. Under drought stress, the expression levels of *CsCOMT6* and *CsCOMT19* showed a decreasing trend with prolonged drought time, and the expression level of *CsCOMT6* decreased more significantly; *CsCOMT25* showed an increasing trend with prolonged drought time. Under MeJA treatment, *CsCOMT24*, *CsCOMT7*, and *CsCOMT10* first decreased and then increased with the prolongation of treatment time, while *CsCOMT18* showed an increasing trend.

### Expression patterns of *CsCOMTs* in different tissues organ

**Fig. 12:**
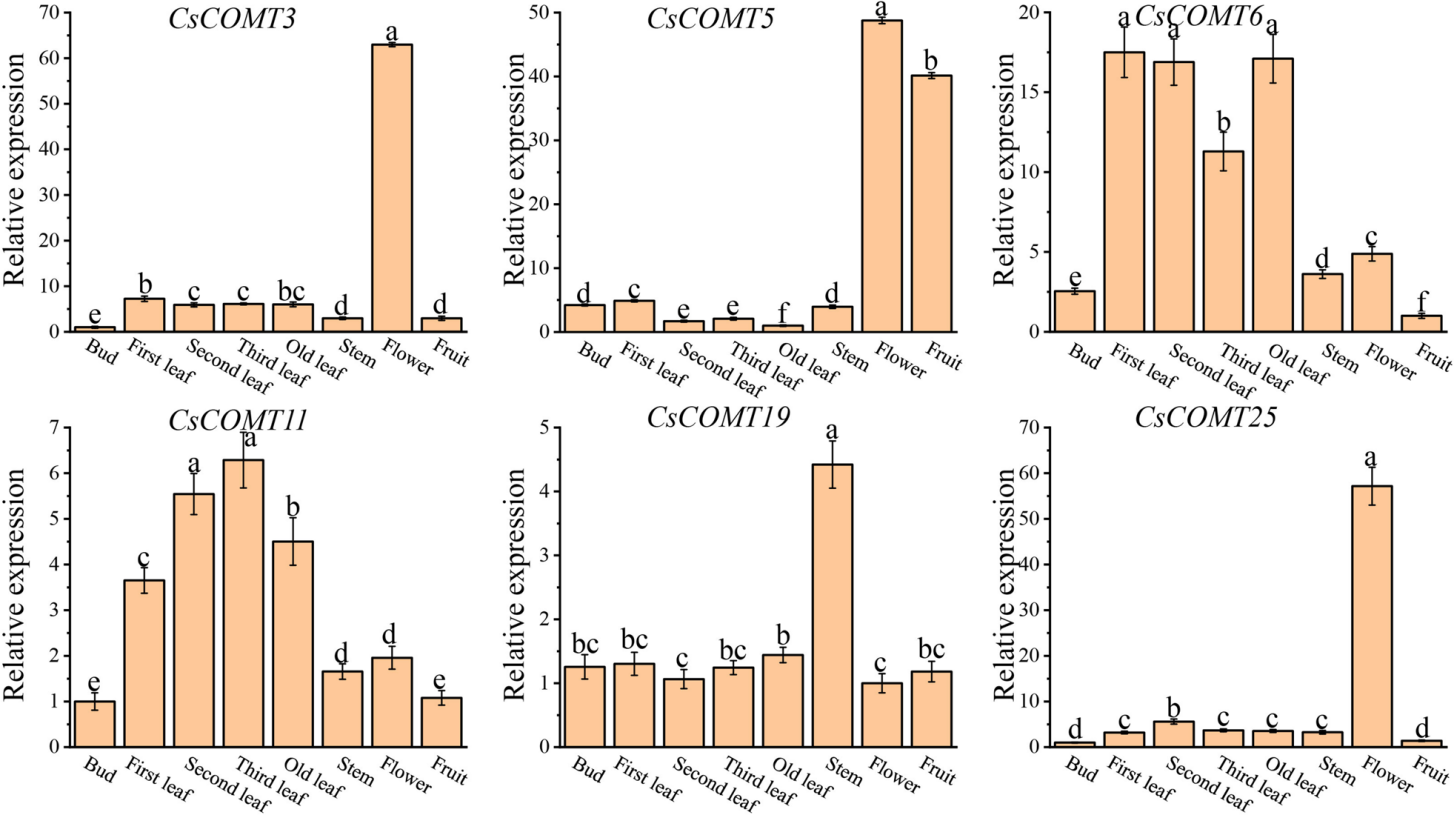
Expression of *CsCOMTs* in different tissues. Different lowercase letters indicate significant differences (*P* < 0.01)

We selected six members of the *CsCOMT* family, including *CsCOMT3*, *CsCOMT5*, *CsCOMT6*, *CsCOMT11*, *CsCOMT19*, and *CsCOMT25* for qRT-PCR to check the expression patterns in different tissues organ (bud, first leaf, second leaf, third leaf, old leaf, stem, flower, fruit) (Fig. 12). The results showed that *CsCOMT3*, *CsCOMT5*, and *CsCOMT25* had relatively high expression levels in all plant tissues organ, with the highest expression levels in flowers. *CsCOMT6* had relatively high expression levels in old leaves, first leaves, and second leaves; *CsCOMT11* had high expression levels in third leaves; and *CsCOMT19* had the highest expression level in stems.

### The effect of *CsCOMT19* on melatonin content in tea plant

**Fig. 13:**
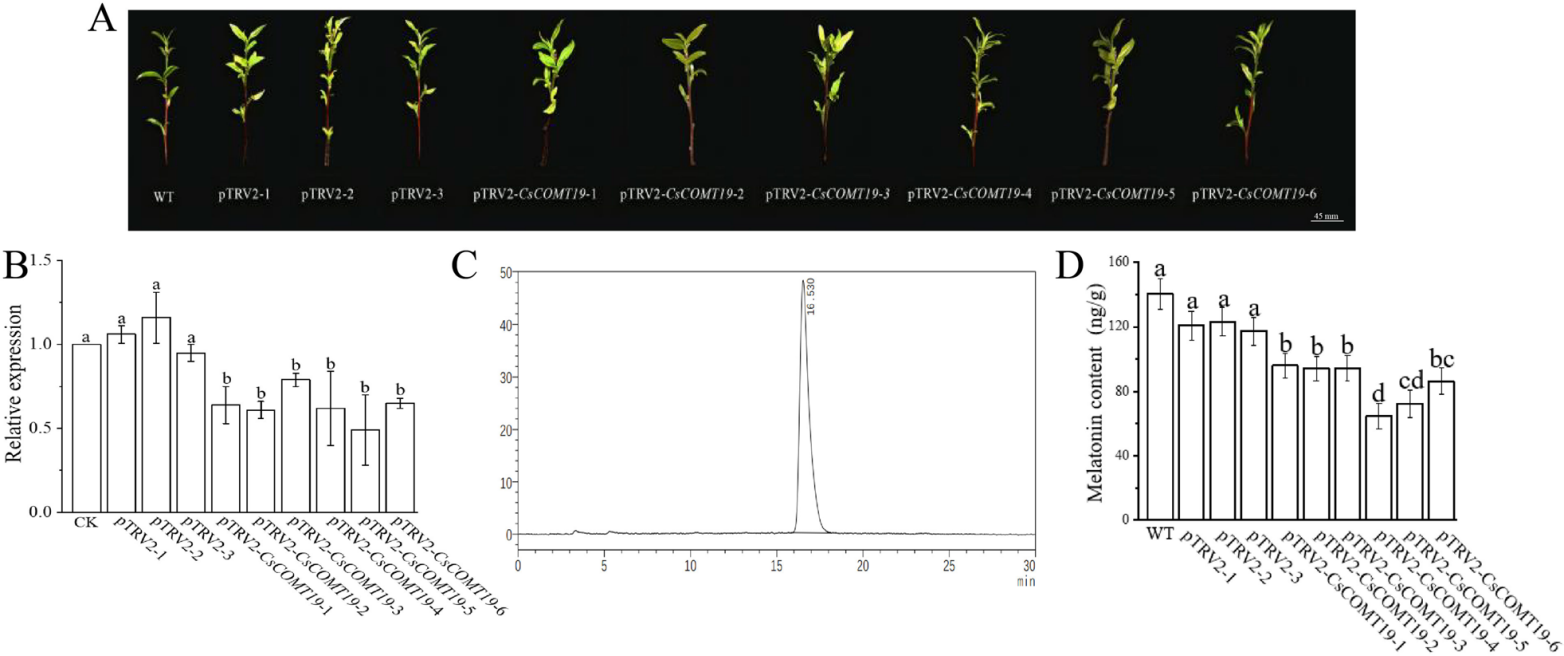
Effects of silencing of *CsCOMT19* on melatonin accumulation in tea plant. **A** Phenotype of *CsCOMT19* silenced tea plant; **B** Chromatogram of melatonin standard (16.530 min); **C** Content of melatonin in control (WT and pTRV2) and *CsCOMT19* silenced plants

*CsCOMT19* has a high expression level in buds and new leaves, and the UTR sequence of this gene is between 300–600 bp, it is suitable for VIGS experiments. Therefore, we constucted *CsCOMT19* gene-silenced plants (pTRV2-*CsCOMT19*) using VIGS method to investigate the effect of down-regulation of *CsCOMT19* expression on the accumulation of melatonin in tea plant (Fig. 13A). After verification by qRT-PCR (Fig. 13B), using HPLC to detect the melatonin content in different types of plants (WT, pTRV2, pTRV2-*CsCOMT19*), it was found that the retention time of the standard sample was 16.530 min (Fig. 13C). In the new buds of WT and pTRV2 plants (control), the melatonin content was 132.17–149.00 ng/g fresh weight (FW) and 117.39–123.12 ng/g FW, respectively. There were no changes in phenotype of *CsCOMT19* gene-silenced plants, but the content of melatonin significantly decreased to a range of 64.70–94.29 ng/g FW, with an average decrease of 41% (Fig. 13D). Thus, it can be seen that there was a positive correlation between the *CsCOMT19* expression and the content of melatonin, indicating that *CsCOMT19* can positively regulate the synthesis of melatonin in the tea plant.

## Discussion

Tea plant is one of the world’s most important beverage crops due to its numerous secondary metabolites conferring tea quality and health effects [24]. The level of stress resistance is an important criterion for evaluating the commercial value of tea plant varieties. The exploration of main-effect genes with higher stress resistance in tea plant has become a research hotspot in tea plant breeding. The *COMT* gene family has a significant impact on plant growth and resistance to stress [6, 25]. With the continuous development of higher plant genome sequencing, *COMT* gene family from many plants were identified, which can regulate plant growth and resistance to stress, such as *Arabidopsis* [11], rice [12], longan [13], etc. However, there are relatively few reports on the *COMT* in tea plant. Therefore, based on the tea plant genome data, we identified and comprehensively analyzed the members of the tea plant *COMT* family (*CsCOMT*).

In this study, a total of 25 *CsCOMT* members were identified from the tea genome (Table 1, Fig. 3), which is significantly different from other species such as *Arabidopsis* (17 members) [11], rice (33 members) [12], and populus (7 members) [26]. Most *COMT* members of tea trees are distributed in the Class I, III, and V families, and they have similar sequences to all members of the *COMT* family in *Arabidopsis*, populus and rice (Fig. 1). The *COMT* family members of tea trees are missing in Class II and Class IV, which may be related to gene replication. Gene duplication probably contributes to the evolution of species and to the adaptation of species to their environments [27]. The results also showed that *CsCOMTs* were mainly located on chromosome 5. In terms of gene structure, most *CsCOMT* family members contained few introns (Fig. 4). Studies have shown that contained few introns was more conducive to plant adaptation to environmental changes [28]. The loss of introns in the *CsCOMT* gene family may be a specific mechanism accompanying evolution, which may promote tea plants to adapt more quickly to stressful environments.

In order to gain a deeper understanding of the evolutionary characteristics of the tea plant’s *CsCOMT* family, a collinearity analysis was conducted on the tea plant’s own genome. Gene duplication probably contributes to the evolution of species and to the adaptation of species to their environments [27]. Four segments with high duplication were found in the collinearity analysis, which may have been caused by the self-replication of the same genes or by chromosomal structural variations during the evolutionary process (Fig. 7). Based on this, collinearity analysis was performed on tea plant, *Arabidopsis*, and poplar, revealing 2 and 7 pairs of collinearity, respectively. This result indicates that the *CsCOMT* family has a higher degree of homology in plants and may have similar biological functions. The analysis of the promoter region *cis*-acting elements revealed multiple components related to growth and stress resistance (Fig. 8), suggesting that it plays an important role in the growth and stress response of tea plants [29].

The results of transcriptome data analysis indicate that the expression of the tea plant *CsCOMT* gene family members varies among different parts of the plant, and there are also differences in tissue expression levels among different members (Fig. 10). For example, *CsCOMT19*, *CsCOMT3*, *CsCOMT25*, and *CsCOMT11* show high expression in various tissues of the tea plant, and these genes may play a critical regulatory role in tea plant growth. When plants were stressed or treated with external hormones, the expression level of *COMTs* increased [30–32]. The above results indicate that the *CsCOMT* family is widely involved in non-biological stress responses and growth and development processes in tea plants. In future research, we can focus on these gene members, which is of great significance for exploring their potential biological functions.

Melatonin is an important signaling molecule with various physiological functions in plant responses to abiotic and biotic stresses [33]. *COMT* involved in melatonin biosynthesis as a key enzyme [10, 11]. In this study, we further investigated the role of *CsCOMT19* in melatonin biosynthesis due to qRT-PCR analysis showed that it was expressed in various tissues organ. The results showed that melatonin synthesis decreased significantly after *CsCOMT19* silencing, indicating that *CsCOMT19* was positively correlation with melatonin content in tea plants.

## Conclusions

Here, this study identified and preliminarily predicted the functions of the *CsCOMT* gene family in tea plant. Our results suggest that the *CsCOMTs* may play important regulatory roles in tea plant growth and stress response. In addition, *CsCOMT19* can positively regulate the synthesis of melatonin in tea plant. This study lays a foundation for exploring the potential applications of the *CsCOMTs*, provides a basis for in-depth understanding of the biological functions of the *CsCOMTs*, and offering information for future tea plant breeding.

## Materials and methods

### Identification and sequence analysis of *CsCOMT* genes

Using the already identified COMT protein sequences from *Arabidopsis* and rice as target sequences to search the *CsCOMT* members from the tea plant genome database (TPIA) by BLAST search tool with an E-value threshold set to be less than 1e-5. Then, the obtained CsCOMT protein sequences were further analyzed for conserved domains using NCBI Conserved Domains Search tool (https://www.ncbi.nlm.nih.gov/Structure/cdd/wrpsb.cgi). Expasy website (https://www.expasy.org/resources/compute-pI-mw) was used to analyze the molecular weight, isoelectric point, amino acid length of CsCOMT [34]. The subcellular localization of CsCOMT was predicted by using pattern recognition database (http://www.csbio.sjtu.edu.cn/bioinf/plant-multi) [35]. Large-scale duplication analysis of *COMT* family members from tea plant, *Arabidopsis*, and poplar draw circos diagrams was performed using TBtools [36].

### Phylogenetic and conserved motif analysis of *CsCOMT* genes

The phylogenetic tree on *CsCOMTs* with *COMTs* from rice, *Arabidopsis*, and poplar was constructed using MEGA 7.0 software with the neighbor-joining method (Boostrap = 1,000). The gene structure of *CsCOMTs* were analyzed by GSDS 2.0 (http://gsds.cbi.pku.edu.cn). MEME (http://memesuite.org) was used to analyze the conserved sequences of CsCOMTs. The amino acid polarity and secondary structure of CsCOMTs were predicted by using PSIPRED [37]. SWISS-MODEL was used to predict the CsCOMTs tertiary structure [38]. The 2000 bp sequences of the translation start sites of *CsCOMTs* was obtained from tea plant genome database, and PlantCARE was applied to predict *cis*-elements of *CsCOMTs* [39]. GO enrichment analysis through TPIA database.

### Transcriptome data analysis

Based on the identified *CsCOMTs*, download their transcriptome data that including different tissues (flower, stem, root, apical bud, young leaf, mature leaf, old leaf), salt stress, PEG-induced drought stress, and methyl jasmonate (MeJA) treatment from the TPIA database (http://tpia.teaplant.org). Use TBtools to construct a heatmap for visualization.

### Plant materials and sampling

Tea plant ‘Fuding Dabai’ from the germplasm resource nursery of the Tea College at Guizhou University was used as experimental materials. One leaf, two leaves, three leaves, old leaves, buds, flowers, stems, and fruits were harvested and immediately frozen in liquid nitrogen, then stored in -80 ℃ freezer for later use.

### VIGS-mediated gene silencing of *CsCOMT19* in tea plant

VIGS technology was used to silence of *CsCOMT19* in tea plants according to the method previously described [40]. In brief, a 292 bp fragment of *CsCOMT19* was assembled into the pTRV2 vector to construct the pTRV2-*CsCOMT19* for VIGS. The pTRV1, pTRV2, and pTRV2-*CsCOMT19* constructs will then be separately transformed into the *Agrobacterium tumefaciens* strain GV3101. After cultivation and resuspension, the *A. tumefaciens* carrying pTRV1 and either pTRV2 or pTRV2-*CsCOMT19* were vacuum infiltrated into tea plant cuttings. The inoculated cuttings was kept in the dark for 3 d, then grown in a greenhouse at 25 ℃ with 16 h light / 8 h dark cycle. This study selected 3 empty vector plants and 6 plants with reduced *CsCOMT19* gene.

### Determination of the melatonin content

Grind the fresh sample and use ultrasonic extraction method. Using a high-performance liquid chromatograph (Waters) with a fluorescent detector, Sunfire C_18_ column (Waters, 4.6 × 150 mm) was employed. The column was gradient eluted for 18 min with a 42%-55% methanol solution in 0.1% formic acid aqueous solution at a flow rate of 0.6 mL/min with an injection volume of 10 μL. The excitation wavelength was set at 280 nm and the emission wavelength was set at 348 nm. The Agilent 1100 liquid chromatograph was coupled to the 6210 TOF electrospray ionization mass spectrometer with a photodiode array detector. The same column and mobile phase were used, with A being methanol and B being 0.1% formic acid aqueous solution. The gradient elution was as follows: A phase increased from 42 to 55% within 18 min, and then decreased to 42% within 1 min. The flow rate was set at 0.6 mL/min and the column temperature was maintained at 25 ℃. The ionization mode was positive and the nebulizer pressure was set at 45 psi. The drying gas flow rate was set at 12 L/min and the drying gas temperature was set at 35 ℃. The melatonin content was quantified accordinng to the melatonin standard curve, and expressed as ng/g FW.

### RNA extraction and quantitative real-time PCR (qRT-PCR)

Total RNA was extracted from the harvested samples using the modified CTAB method, then the cDNA was synthesized using the reverse transcription kit (Genenode, Wuhan, China) according to the manufacturer’s instructions. The primers used for gene expression alanysis were designed using Primer Premier 6.0 software (Table S2). The *Actin* was used as the internal reference gene. qRT-PCR was carried on a CFX100 Realtime PCR System (Bio-Rad, CA, USA). The reaction conditions and system were performed according to the SYBR Green Realtime-PCR Mastermix instructions, and the results were calculated using the relative quantification 2^−ΔΔCT^ method [41]. There are three treatments each time, and each treatment has three plants.

### Statistical analysis

Excel 2010 was used to organize and analyze statistical data. The data statistical significance was analyzed by one-way and two-way ANOVA in SPSS 19.0, with the LSD method at the significance level at *P* < 0.05.

### Supplementary Information

**Additional file 1: Fig. S1.** Number of *COMT* families in the *C.sinensi*, *A.thaliana*, *O.sativa* and *P.trichocarpa*, and the proportion of the total number of 104, respectively. **Fig. S2.** Number of *COMT* families in the different groups. **Fig. S3.** The sequences of 15 motifs of *CsCOMT* in tea plant. **Table S1.** The distribution ratio of *CsCOMT* genes on each chromosome in *C.sinensis*. **Table S2.** Primer sequence for qRT-PCR.

## Data Availability

The datasets generated and/or analysed during the current study are available in the TPIA database, http://tpia.teaplants.cn/index.html.
