# Supplementary material for: Genome-wide characterization of COMT family and regulatory role of CsCOMT19 in melatonin synthesis in Camellia sinensis
Source: BMC Plant Biol. 2024 Jan 16;24:51. doi: 10.1186/s12870-023-04702-0 (PMC10790539; doi:10.1186/s12870-023-04702-0)
Supplement: Supplementary file 1 — Additional file 1: Fig. S1. Number of COMT families in the C.sinensi, A.thaliana, O.sativa and P.trichocarpa, and the proportion of the total number of 104, respectively. Fig. S2. Number of COMT families in the different groups. Fig. S3. The sequences of 15 motifs of CsCOMT in tea plant. Table S1. The distribution ratio of CsCOMT genes on each chromosome in C.sinensis. Table S2. Primer sequence for qRT-PCR. [file 12870_2023_4702_MOESM1_ESM.doc]

**Genome-wide characterization of *COMT* family and regulatory role of *CsCOMT19* in melatonin synthesis in *Camellia sinensis***

Thanh Huyen Pham ^a^, Xingyu Tian ^a^, Huimin Zhao ^a^, Tong Li ^b^*, Litang Lu ^a,b^*

^a^ College of Life Science, The Key Laboratory of Plant Resources Conservation and Germplasm Innovation in the Mountainous Region (Ministry of Education), Guizhou University, Guiyang, 550025, People’s Republic of China

^b^ College of Tea Science, Guizhou University, Guiyang, 550025, People’s Republic of China

*Correspondence

Tong Li :lit@gzu.edu.cn; Litang Lu: [ltlv@gzu.edu.cn](mailto:ltlv@gzu.edu.cn)


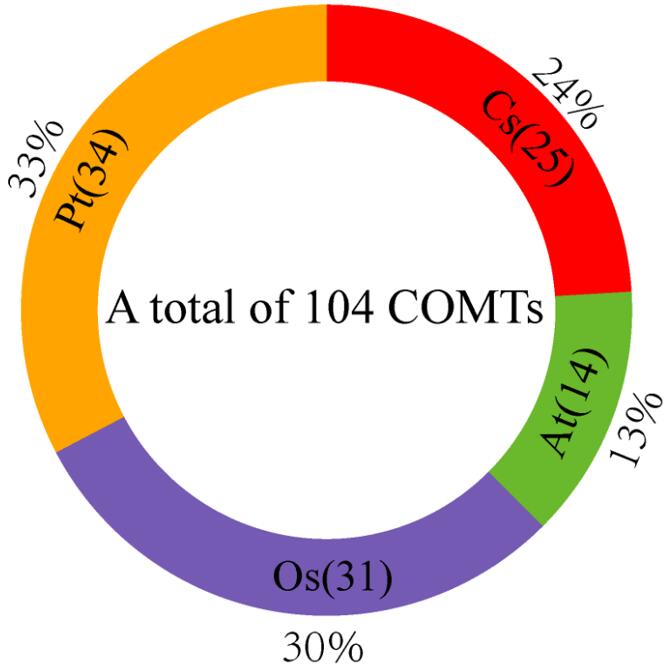


**Fig. S1** Number of *COMT* families in the *C.sinensi*, *A.thaliana*, *O.sativa* and *P.trichocarpa*, and the proportion of the total number of 104, respectively


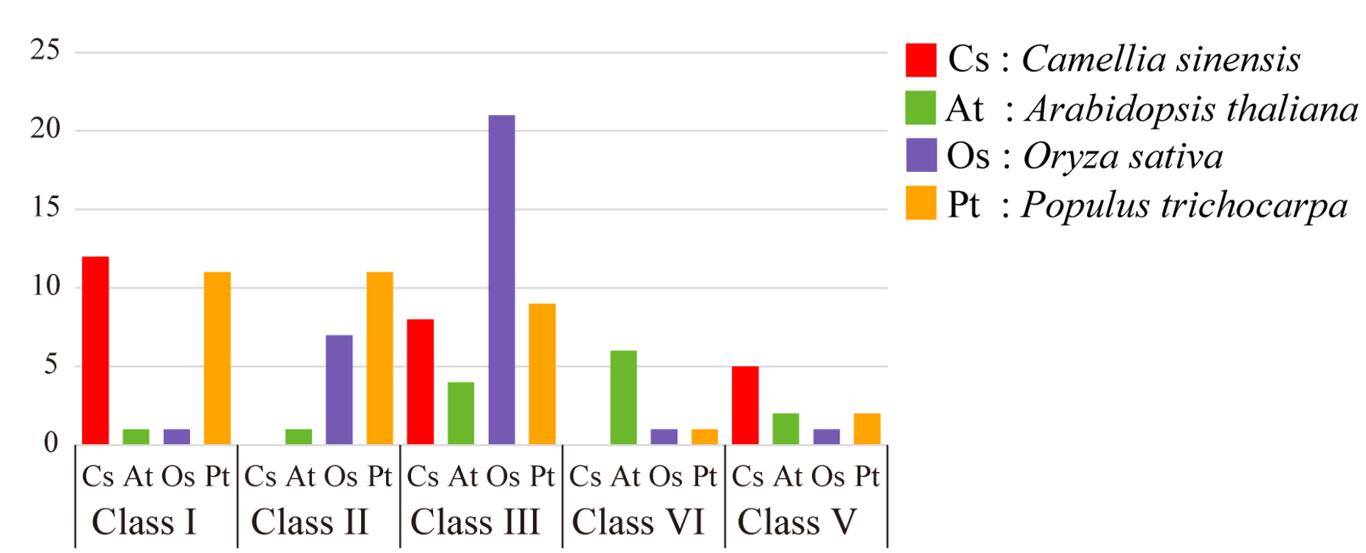


**Fig. S2** Number of *COMT* families in the different groups


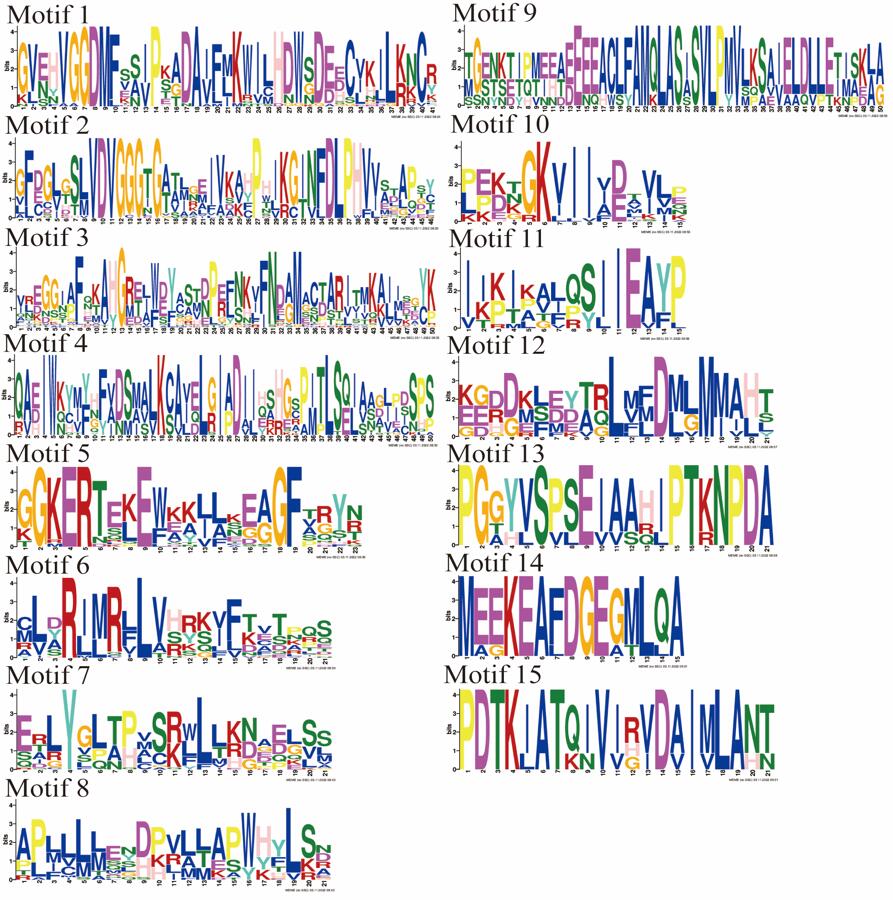


**Fig. S3** The sequences of 15 motifs of *CsCOMT* in tea plant

**Table S1** The distribution ratio of *CsCOMT* genes on each chromosome in *C.sinensis*

| Chr | Number of genes | Number of *CsCOMT* in *C.sinensis* | Percentage of *CsCOMT* in *C.sinensis* |
| --- | --- | --- | --- |
| Chr1 | 3849 | 1 | 0.02598% |
| Chr2 | 3814 | 2 | 0.05243% |
| Chr3 | 3258 | 5 | 0.15346% |
| Chr5 | 2843 | 7 | 0.24622% |
| Chr6 | 3332 | 3 | 0.09000% |
| Chr7 | 3301 | 5 | 0.15146% |
| Chr8 | 2237 | 1 | 0.04470% |
| Chr | 2737 | 1 | 0.03653% |

**Table S2 Primer sequence for qRT-PCR**

| **Gene** | **Forward primer (5'-3')** | **Reverse primer** **(5'-3')** |
| --- | --- | --- |
| *Actin* | CAGACCGTATGAGCAAGGAAAT | GTGCTTAGGGATGCAAGGATAG |
| *CsCOMT3* | CAAGGTGTTCAACCAAGGAATG | CGTCGAAGCCTCCGTAAATATC |
| *CsCOMT5* | ATTCCCACTCGAAATCCTGAC | GCTGCCGCTGCTATTATTTATG |
| *CsCOMT6* | CGGAGATAACGTCTCGTCTAGTA | GTATGAGCAGAGCAAGTGAGAA |
| *CsCOMT11* | GCCTGAACCACTGCTCTAAA | CATCGCTCCAATCATGCAATATC |
| *CsCOMT19* | GAACCAAGGCTGAACCATTTC | GGTCTTGGAGAGTACAGTTGAG |
| *CsCOMT25* | GCGTCTGTTCTTCCCTATGT | GGTCTTGGAGAGTACAGTTGAG |
